# Using the *Drosophila* Nephrocyte to Model Podocyte Function and Disease

**DOI:** 10.3389/fped.2017.00262

**Published:** 2017-12-07

**Authors:** Martin Helmstädter, Tobias B. Huber, Tobias Hermle

**Affiliations:** ^1^Renal Division, University Medical Center Freiburg, Freiburg, Germany; ^2^Faculty of Medicine, University of Freiburg, Freiburg, Germany; ^3^III. Department of Medicine, University Medical Center Hamburg-Eppendorf, Hamburg, Germany

**Keywords:** nephrocyte, *Drosophila*, podocyte, glomerular disease, garland cell, endocytosis, renal disease

## Abstract

Glomerular disorders are a major cause of end-stage renal disease and effective therapies are often lacking. Nephrocytes are considered to be part of the *Drosophila* excretory system and form slit diaphragms across cellular membrane invaginations. Nehphrocytes have been shown to share functional, morphological, and molecular features with podocytes, which form the glomerular filter in vertebrates. Here, we report the progress and the evolving tool-set of this model system. Combining a functional, accessible slit diaphragm with the power of the genetic tool-kit in *Drosophila*, the nephrocyte has the potential to greatly advance our understanding of the glomerular filtration barrier in health and disease.

## Introduction

Disorders that affect the glomerulus are the predominant cause of end-stage renal disease (ESRD) (1). Beyond renal replacement therapy, potent therapeutic options are not available for most of the disorders from this heterogeneous group. This underscores the unmet need for valid model systems that serve to understand the mechanisms of glomerular diseases.

The most established model system for the glomerulus is currently the mouse. Despite being the undeniable gold standard, this model has its inherent limitations in costs, speed, and considerations of animal welfare. Complementary systems are thus desirable. An excellent alternative can be found in the zebrafish model. But the aforementioned limitations of the mouse model are alleviated only in parts in zebrafish, while additional obstacles like the shortcomings of the morpholino technology need to be considered. Significant insights were also derived from *in vitro* studies, mainly by using cultured podocytes (2). However, here other significant limitations are arising. This is mainly a consequence of the complex glomerular architecture. The glomerular filter is three-layered, including the fenestrated endothelium, the glomerular basement membrane, and the podocytes that form the slit diaphragm. Podocytes are characterized by their most intricate cell shape that is paralleled only by neuronal cells. Cultured podocytes lack crucial features of their *in vivo* counterparts, most notably the formation of slit diaphragms. The *Drosophila* anatomy contains no structure whose analogy to the mammalian kidney appears obvious. Thus, it was unexpected that the *Drosophila* nephrocyte was identified as an invertebrate model that has the potential to meet the needs for a complementary model for glomerular disease (3, 4).

Discovered more than 150 years ago (5) and identified as storage kidneys, the nephrocyte received limited attention until after the discovery that their auto-cellular junctions need to be considered as slit diaphragms that are formed by the orthologs of the mammalian slit diaphragm proteins nephrin (*sns*) and *NEPH1 (kirre)* (3, 4). Loss-of-function of slit diaphragm proteins results in a smooth cell surface analogous to podocyte foot process effacement. Nephrocytes thus share molecular, ultrastructural, and functional features with podocytes (3, 4, 6, 7). This makes nephrocytes a unique model system with a functional slit diaphragm in a genetically highly tractable model organism. Now, nearly a decade after the introduction of the nephrocyte to a wider audience in the renal field, the purpose of this review is to examine the progress and the evolving tool-set of this model system for glomerular diseases that still has not reached its peak.

## Nephrocyte Biology

Regarding the history and basic principles of nephrocyte biology, we refer to detailed previous reviews (8–10) in order to focus on the more recent findings.

### Basic Concepts of Nephrocyte As an “Excretory” Organ

**Figure 1:**
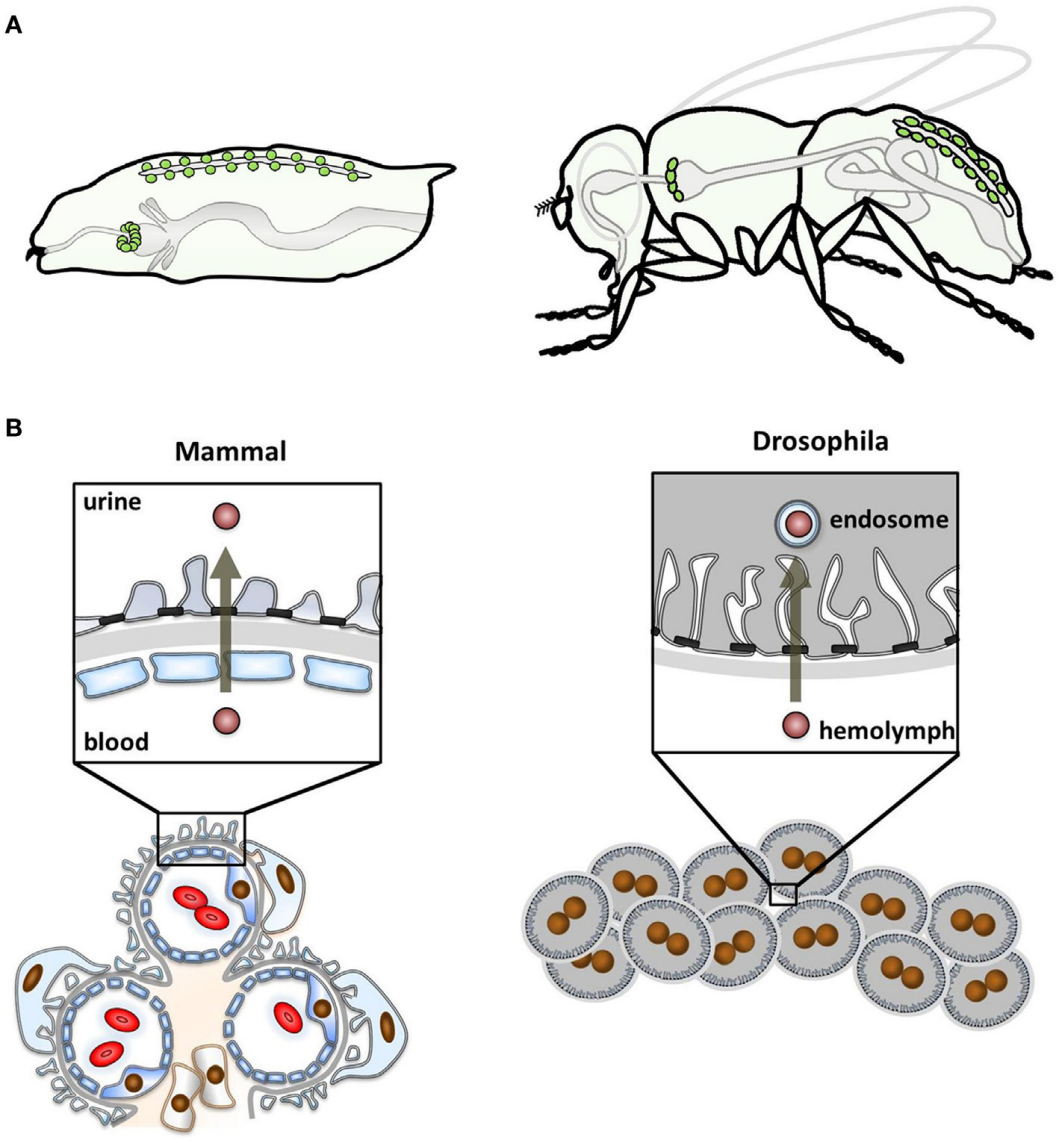
Localization of nephrocytes and functional comparison with mammals. **(A)** The schematic shows the anatomical position of nephrocytes (green) within the larval body (left) and the adult fly (right). Garland cell nephrocytes localize within a ring around the esophagus and pericardial nephrocytes are found in pairs along the heart tube. **(B)** The schematic compares the glomerular function of mammals (left) and *Drosophila* (right). The mammalian glomerular filter is three-layered, consisting of the fenestrated endothelium, basement membrane, and the podocytes that form foot processes. Between these, the slit diaphragm is localized. Filtration occurs from the blood through the filter into the urinary space. In *Drosophila*, nephrocytes function as individual cells that are clustered. The glomerular filter is bi-layered, lacking the endothelium. Filtration occurs from the larval blood, the hemolymph, being destined for processing in endosomes.

The two fundamental functional subunits of renal organs throughout vertebrate biology are the glomeruli that produce an ultrafiltrate and the tubules that process the ultrafiltrate further and finally feed the resulting urine into the disposal system. Together, they serve to eliminate toxins and waste products and maintain water, salt, and pH homeostasis. Following similar principles, *Drosophila* nephrocytes are considered to be part of the fly excretory system. The *Drosophila* renal system has two functional subunits as well: the nephrocytes, regarded as analogous to the glomeruli, and the Malpighian tubules, regarded as analogous to the renal tubular system. There are two distinct nephrocyte populations: the pericardial nephrocytes along the heart tube and the garland cell nephrocytes in a garland-like ring around the esophagus (Figure 1A). However, a substantial conceptual difference to the mammalian kidney is that nephrocytes have no connection to the tubular system of *Drosophila* that independently generates, modifies, and finally excretes urine into the intestinal lumen (8).

### Brief History of Nephrocyte Research

**Figure 2:**
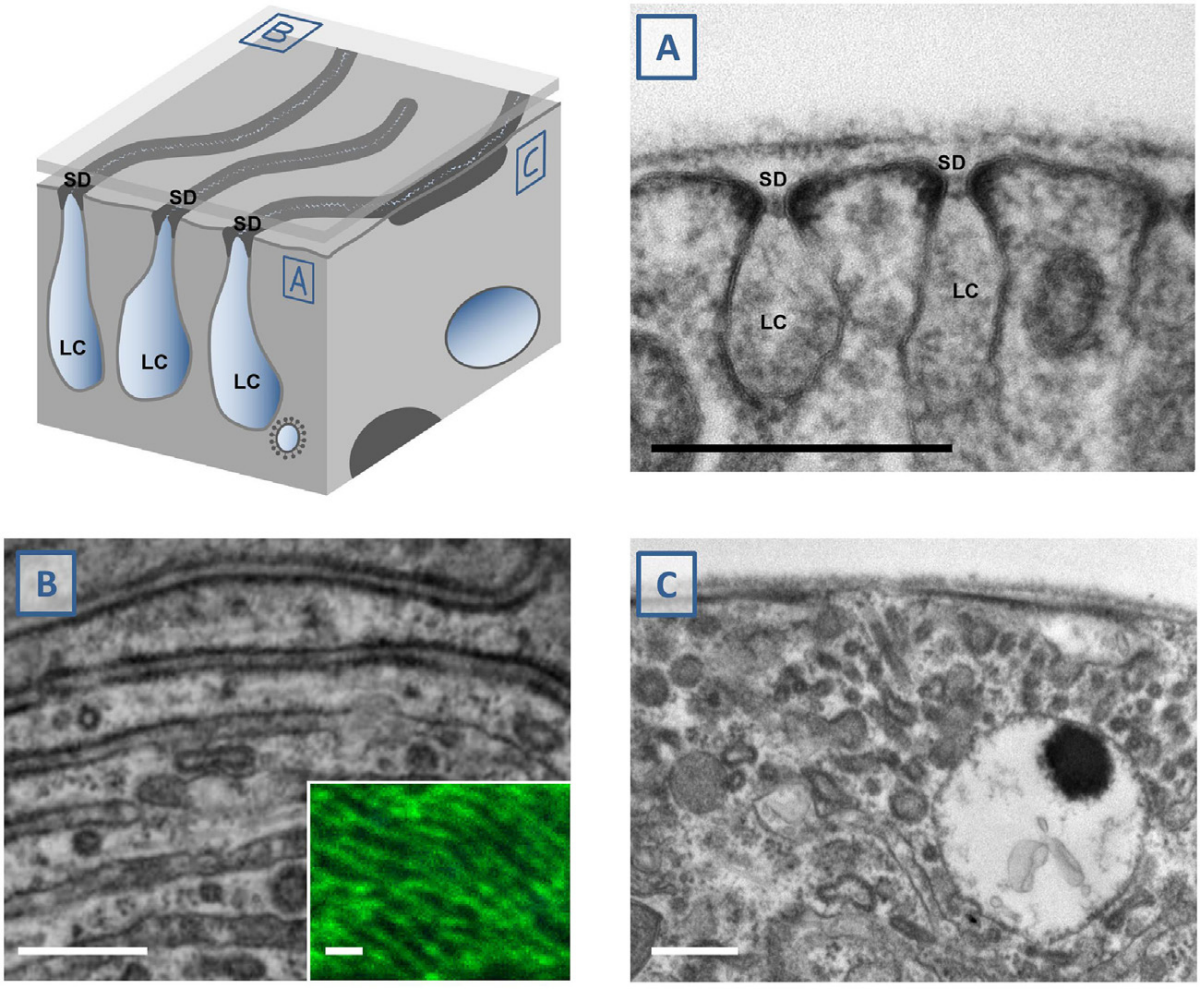
Study of nephrocyte ultrastructural morphology using electron microscopy. The schematic illustrates a surface detail of a nephrocyte. Shown are labyrinthine channels as membrane invaginations that are sealed by the slit diaphragm. **(A)** The analogy to podocyte ultrastructure is most obvious in sections that are perpendicular to the nephrocyte surface. The slit diaphragm is recognizable as a fine line next to the electron-density around the entry to the membrane invagination. **(B)** Tangential sections through the surface show the slit diaphragm as continuous lines. This fine linear pattern correlates with the findings by confocal microscopy and staining of the ortholog of nephrin, sns (inset). **(C)** Perpendicular sections through the nephrocyte surface may be confusing when they cut through the nearly parallel lines of the slit diaphragms at an oblique angle. This results in an image that makes it difficult to identify the slit diaphragms and labyrinthine channels. Stretches of electron-density below the surface are mere indicators of their presence. SD indicates slit diaphragm, LC indicates labyrinthine channels, scale bars represent 500 nm.

Despite the divergence of anatomy and functional concepts, the role of nephrocytes was already discovered in the mid-19th century. The garland cell nephrocytes were first described in 1864 by August Weismann in Freiburg. Studying larvae from *Musca vomitoria*, he noted a string of cells reminiscent of a garland that was floating in the larval body cavity. Accordingly, he termed them “Guirlandenzellen” (German *Girlande* means garland) (11). The function of these cells was an utter mystery to their discoverer but not much later, in 1886, Kowalevsky intended to stain the intestinal mucosa of muscid larvae by feeding them carmine, methylene blue or silver. However, he noted that only nephrocytes took up and stored these tracers (12). He correctly concluded from the tracer experiments that these cells function like a storage kidney to clear the larval blood. This concept was later expanded by Hollande (13). The elaborate ultrastructure of *Drosophila* nephrocytes was described in the sixties of the last century (14, 15) and few years later Crossley (16) showed the size-selectivity of uptake and already noted the analogy of this ultrastructure to podocytes: membrane invaginations called labyrinthine channels are bridged by an auto-cellular slit diaphragm that controls their entry. Nephrocytes are covered by a basement membrane. Thus, two of the three layers of the glomerular filter in vertebrates are found in *Drosophila* nephrocytes (Figures 1B and 2A).

After the discovery of nephrin (17), Skaer (3), Abmayr and colleagues (4), finally put the pieces together and proved a molecular analogy of podocytes and nephrocytes. They noted that the orthologs are both expressed in nephrocytes and that their absence results in nephrocyte loss of the complex invaginations and a smoothing of the cell surface (3, 4). Interestingly, this disruption of nephrocyte ultrastructure also follows treatment with puromycine (18) and protamine sulfate (7), both of which have the same effect in podocytes. Mammalian NEPH proteins have been shown to be functional within this invertebrate system (19). A most recent and surprising discovery is that nephrocytes potentially exhibit apico-basal polarity (20). Crumbs, a marker of apical polarity, was observed along the membrane of the labyrinthine channels. The presence of integrins on the nephrocyte surface may be suggestive of a baso-lateral compartment (7). This raises the possibility that despite being spherical cells, nephrocytes might be polarized within their complex ultrastructural morphology. Consequently, analogous to mammalian podocytes, apico-basal polarity may be a potential prerequisite for nephrocyte morphology and function (20). However, at this point it has not been ruled out that the functional role of these proteins in nephrocytes is independent from polarity.

### Endocytosis As an Essential Nephrocyte Function

It is astounding that nephrocytes can exert their function as an “excretory” organ without a connection to the outside world. This feat is performed by endocytosis. The first step is endocytic uptake of material from the blood of the fly that is called hemolymph. Nephrocytes then sort the endocytic cargo for either degradation in the lysosome or recycling back to the hemolymph. Unwanted cargo that cannot be destroyed, will be stored intracellularly. In that way, nephrocytes detoxify the hemolymph and nephrocyte dysfunction reduces survival upon toxin exposure (3). Nephrocytes thus do not contribute to urine formation in any way but nevertheless the high endocytic activtity can be regarded as “excretory” activity (Figure 1B). To be optimized for that purpose, they increase their surface area by forming invaginations from the cell surface called labyrinthine channels. Endocytic uptake may occur from the cell surface but the major site of endocytosis is from within the labyrinthine channels (21, 22). These regularly spaced furrows cover the entire nephrocyte surface (4, 7). The high endocytic activity attracted researchers to use nephrocytes as a model to study mechanisms of endocytosis (22–26). Endocytic uptake is at least in part dependent on the ortholog of Cubilin (7, 27, 28). In face of this conceptual similarity to the proximal tubular function, it has been proposed that nephrocytes are also a model for the proximal tubule (28). Nephrocytes have been shown to regulate the hemolymph composition through their endocytic activity (29).

### Developmental Aspects

Like podocytes, both subsets of nephrocytes are derived from the mesoderm. Differentiation and maintenance of nephrocytes requires *dKLF15* as a nephrocyte-restricted growth factor (30). *dKLF15* is the ortholog of Krüppel-Like Factor 15 which plays a role in podocyte development (31). The expression of *sns* and *kirre* and the formation of slit diaphragms begin during mid-embryogenesis and are maintained through larval development into adulthood (3, 4, 7, 32). Garland cell nephrocytes undergo a fusion process between embryonic stage E13 and E18, resulting in binucleate cells (4). For unknown reasons, the cell number decreases for both, garland cell and pericardial nephrocytes throughout development. About a quarter of the 25 garland cell nephrocytes and 120 pericardial nephrocytes that are present at the end of embryogenesis are maintained during development into adulthood (4, 7, 8, 33). Adult garland cell nephrocytes exhibit tracer uptake and show regular ultrastructural morphology, supporting these to be functional cells (7).

### Frontiers of Nephrocyte Research

Despite the considerable interest nephrocytes have gained in the recent past, we do not fully understand the significance of nephrocytes within the adult fly organism. Ivy et al. (30) recorded the life-span of adult flies that lack nephrocytes due to the absence of *Klf15* in comparison with wild-type. In contrast to the observations in larval stages, the absence of nephrocytes did not affect survival of adult flies, not even under toxin stress. Further work will be required to determine the functional role of nephrocytes in the organism of adult flies.

A secretory function of nephrocytes that is suggested by the abundance of rough endoplasmic reticulum has been shown for lysozyme (16) but this functional aspect has not been further explored.

Regarding the two subsets of nephrocytes, i.e., pericardial and garland cell nephrocytes, it has become overwhelmingly clear that both cells share critical features. This includes gene expression patterns of the slit diaphragm proteins and other genes like *dKlf15, hand, rudhira*, or *Amnionless*. On the other hand, obvious differences define them as distinct cell lineages, beginning with the number of nuclei. These differences are still not explored in detail. Currently, garland cell and pericardial nephrocytes are best considered to be complementary.

## Readouts and Strategies of Nephrocyte Research

The functional tool-set for nephrocytes is still evolving. As a consequence, there is no defined optimal strategy or established gold standard. However, a general rule emerges that a functional and a morphological assay need to be combined. We will discuss the merits and pitfalls of different strategies to give an overview.

### Tracer Endocytosis As a Readout of Nephrocyte Function

Tracer endocytosis stood at the very beginning of nephrocyte research when they enabled Kowalevski to identify clearance of the larval circulation as their functional role. Until present time, virtually any manuscript regarding nephrocytes employs tracer uptake as a functional readout. This is based on the fact that the “excretory” function of nephrocytes occurs *via* endocytosis. An analogous effect has also been shown for endogenous proteins like *imaginal disk growth factor 2* (34).

A broad array of tracers has been used since Kowalevsky, who introduced AgNO3 that is still in use today. The most commonly used tracers are proteins like GFP-derivates (6), albumin (7), avidin (26), wheat germ agglutinin (22), or Horseradish peroxidase (HRP) (16). Another common approach uses polysaccharids like dextrans (3). Less commonly employed are colloidal substances like Coomassie Brilliant Blue (35). The choice of tracer may follow personal preference but it is important to consider the basic principles for these experiments that became clear through recent findings.

The broad range of possible tracer substances is astonishing. This reflects that the uptake of at least some tracers occurs *via* the scavenger receptor Cubilin (7), whose mammalian ortholog shows affinity for a broad range of ligands as well (36). Aside from the drastic reduction of tracer endocytosis upon silencing of Cubilin/Amnionless, this concept is supported by experiments that suggest that tracer uptake occurs receptor-mediated (7). The observation by Crossley (16) that Lanthanum-dioxide and HRP both enter the labyrinthine channels, while only the latter is taken up, indicates a certain extent of selectivity for the entry mechanism which most likely reflects the receptor affinity.

Being dependent on the scavenger receptor, measurements of nephrocyte tracer endocytosis for most tracers thus do not simply reflect the surface area of nephrocytes. The interpretation of experiments applying tracer endocytosis in nephrocytes should always take possible effects on receptor abundance, function, or specificity into account.

Filtration of tracers across the nephrocyte slit diaphragm is an important concept that directly relates to the functional analogy to podocytes and thus also underlines the significance of such experiments. Crossley (16) was able to show that in nephrocytes, tracers may be excluded size-dependently by the basement membrane and the slit membrane, thus defining a cut-off of about 12 nm. More recently, receptor competition experiments revealed that this size cut-off is comparable with the mammalian glomerulus around 70 kDa (7). Basement membrane thickening occurring upon loss of slit diaphragm proteins (3) mainly affects tracers with a high molecular weight while this effect is not directly related to the nephrocyte ultrastructure or filtration. This became obvious when it was shown using a rapid intervention like protamine treatment that perturbs the ultrastructure without thickening the basement membrane. This treatment has no effect on tracers with a high molecular weight while a tracer that is below the filtration cut-off is strongly reduced (7). In light of these findings, a tracer that is smaller than 70 kDa appears to be preferable. Another potential confounding effect comes with the saturability of tracer endocytosis (7). Choosing an excessive tracer concentration or incubation period thus may even blur the effect of silencing *sns* despite using tracers that are small enough to pass the slit diaphragm (3).

The two major alternative strategies are a pulsed uptake by exposing nephrocytes to tracers *ex vivo* or using an endogenous tracer *in vivo*. The latter strategy was first described by Han and colleagues (6). It employs a transgenic fusion protein of red fluorescent protein with the atrial natriuretic factor from rat. This transgenic tracer is expressed endogenously by muscle cells, secreted into the larval circulation followed by endocytosis and degradation by nephrocytes. The fluorescence intensity of larval pericardial nephrocytes can be observed by transcutaneous imaging. This elegant approach is fast and enabled large-scale genetic screens (6). Studying nephrocytes *in vivo*, this approach appears superior to *ex vivo* interventions. On the other hand, one has to be conscious of certain limitations that nevertheless may favor a decision for an *ex vivo* setting. Imaging fluorescence through the larval cuticle may entail weak and variable signal intensity. True *in vivo* imaging is complicated by larval movements that usually require measures to reduce larval motility. The continuous tracer expression furthermore inherently renders the net result from uptake and degradation as readout. A block in degradation and an increase in uptake thus may have the same outcome. Possible variations in tracer production, distribution, and alternative degradation are difficult to control. The *ex vivo* incubation on the other hand, is able to exclusively reflect rapid uptake under controlled conditions with direct imaging of the nephrocytes. These experiments do not require the presence of a transgene that controls the expression of the endogenous tracer and thus can be immediately applied in any genetic background that allows larval survival. This approach further allows the combination with additional interventions like drug exposure. The dissection of garland cell nephrocytes can be accomplished within few seconds which minimizes the impact of an *ex vivo* setting. An intermediate strategy by dissecting nephrocytes from transgenic animals that express a tracer endogenously may allow to combine some of the advantages of both approaches like speed and precision of direct imaging. Then again, this also retains disadvantages from both sides like the confounders entailed by continuous tracer production and the invasiveness of dissection. In summary, application of either strategy needs to be tailored to the experimental question and the available resources.

Finally, an important consideration for any uptake experiment is that it is hard to interpret without additional morphological studies. As we can learn from loss-of-function of endosomal proteins, reduced tracer uptake may occur while the slit diaphragms are maintained (25, 26) (or potentially also *vice versa* in different settings).

### Electron Microscopy As a Readout of Nephrocyte Morphology

The major advantage of nephrocytes lies in the formation of functional slit diaphragms that are easily accessible. These slit diaphragms can be observed directly by transmission electron microscopy (TEM) providing the most unequivocal evidence. That way the ultrastructural analysis is an important complement for the tracer studies whose interpretation can be more difficult but that still are indispensable as a rapid functional assay. Although the technique is well established after many decades of research, study of nephrocytes still holds challenges that entail the complex ultrastructural architecture of these cells. The labyrinthine channels are nearly parallel furrows that are observed across the whole nephrocyte surface (Figure 2). The slit diaphragms seal these invaginations like a ceiling. Tangential sections through slit diaphragms therefore impress as parallel lines (4, 7) (Figure 2B). The interpretation of equatorial cross sections is more difficult, as they are dependent on their orientation in regard to the lines formed by the slit diaphragm. A cross-section that cuts through the slit diaphragms at an approximately perpendicular angle renders the classical image (Figure 2A). However, if the section cuts through the parallel lines of the slit diaphragms at an angle that is too oblique (Figure 2C), the slit diaphragms and labyrinthine channels will be distorted and difficult to identify. For that reason, such a section may even be mistaken for a smoothing of the nephrocyte surface. However, these oblique sections are marked by elongated stretches of electron-density (Figure 2C) that clearly distinguish them from a true phenotype. Unfortunately, examples for this error can even be found in the literature.

Analogous to the observations regarding the mammalian slit diaphragm (37), one can assume that fixation and sample preparation may influence the resulting image.

Scanning electron microscopy (SEM) can be most useful to gather information regarding cell shape and number (4, 19), while this may be achieved by using approaches involving light microscopy as well. The assessment of the nephrocyte ultrastructure through this approach is complicated as the labyrinthine channels are concealed by the basement membrane. Specific protocols like protease treatments can overcome this limitation but need to be considered carefully to avoid artefact.

### Immunofluorescence (IF) As Complementary Strategy

Immunofluorescence of slit diaphragm proteins facilitated the discovery of nephrocytes as a model for glomerular diseases (3, 4). Still, this assay is regarded a complementary strategy that was omitted in a number of manuscripts. A significant recent observation concerns highly resolved tangential confocal sections of nephrocytes stained for Sns or Kirre. These stainings reveal a fingerprint-like pattern that correlates with the picture obtained in tangential sections in TEM (Figure 2A inset) (7). This not only implies that findings obtained with TEM can be backed up by stainings of the slit diaphragm proteins but also that significant information about ultrastructural aberrations can be obtained before applying TEM. Combined with the opportunity of co-IF, this allows a more comprehensive analysis for a number of questions. IF with 3D reconstruction also revealed that the entire cell body is covered by slit diaphragms (7). In a similar fashion, IF can be regarded as an alternative to SEM.

It remains to be seen, if super resolution imaging using stimulated emission depletion microscopy or stochastic optical reconstruction microscopy will be able to render additional information.

### Readouts of Mortality

Observing survival under the exposure to toxins like silver nitrate in larvae has been described as a possible assay that directly relates to nephrocyte function. That way it may be a suitable complement for tracer strategies that documents the effect on the larval physiology. Another assay that has been introduced more recently is to record adult life-span (38). This appears a logical addition to the spectrum of investigations. However, at this point in time, it is difficult to connect reduced adult survival with nephrocyte functions due to the findings by Hartley and colleagues (30). They did not observe an altered life-span when nephrocytes were even entirely absent entirely due to lack of *dKlf15*. Potential confounders for fly survival can result from expression in other tissues. The commonly used *GAL4*-drivers to control expression of RNAi do not follow anatomic structures but are dependent on gene expression patterns. It is very difficult to rule out that temporary or very limited expression in other tissues contributes to a broad compound readout like survival. At least until the role of nephrocytes in the physiology of the adult animal is better understood, in our opinion it appears prudent to rather make use of assays that are more specifically tailored to nephrocytes.

### Loss-of-Function Strategies

The virtually genome-wide availability of cost-effective, off-the-shelf RNAi libraries is a major advantage of *Drosophila* as a model system. Loss-of-function strategies that employ RNAi are inherently limited by potential off-targets or inefficient knockdown. This may result in contradicting results between different RNAi-transgenes. A common strategy in the *Drosophila* field consists in analyzing at least two independent RNAi stocks to confirm the findings. Further confirmation often remains desirable. This can be achieved by utilizing genetic null alleles. However, lethality frequently impedes this strategy like in the case of *sns*. Nephrocytes are not amenable to the classic mosaic analysis in *Drosophila* as they are non-dividing cells. The CRISPR/Cas9 technology allows to introduce a conditional loss-of-function that is not RNAi dependent (7). This can be accomplished by transgenic *Drosophila* that combine nephrocyte-restricted expression of Cas9 in conjunction with ubiquitous expression of a directed guide RNA. In this approach, individual cells may have divergent phenotypes dependent on individual CRISPR/Cas-induced mutations. Possible draw backs of this strategy are off-targets, inefficient gene disruption, and persistence of mRNA. Therefore, it is reasonable to either use two independent guide RNAs or combine this with an RNAi approach. For both methods, RNAi and CRISPR/Cas, loss-of-function best is further verified, e.g., by IF using a specific antibody or rescue experiments with an RNAi/CRISPR-resistant transgene.

A number of different *GAL4*-driver lines have been utilized to control the expression of transgenes to induce loss-of-function in nephrocytes. These include *Dorothy*-*GAL4* (6, 39), *sns*-GCN-*GAL4* (4, 40), and *Hand*-*GAL4* (41). While these lines express *GAL4* in both subsets of nephrocytes, the equally common *prospero*-*GAL4* (3, 42) has predominantly been used for garland cell nephrocytes. A garland cell nephrocyte-restricted expression has been described for *Aug21*-*GAL4* (43). All commonly used driver lines appear to exhibit robust expression during the larval stage. *G447.2-GAL4* (3) was used to direct expression in embryonic garland cell nephrocytes. *Dorothy*-*GAL4* (7, 44, 45), *Hand-GAL4* (46), and *sns-*GCN*-GAL4* (47) have been employed in nephrocytes dissected from adult animals. A systematic comparison of the available *GAL4* lines regarding onset, continuation, and intensity of transgene expression is currently lacking. The same is true for a characterization of the extra-nephrocytic expression that almost certainly occurs in all the *GAL4*-drivers.

## Nephrocyte as Disease Model

### Monogenic Forms of Nephrotic Syndrome

*Drosophila* shows a surprising extent of conservation of disease genes and this model organism has proven to be appropriate for the investigation of monogenic diseases. These disorders are characterized by mutation of a single gene as the molecular cause of a disease. The role of nephrocytes as a model for genetic kidney disease has recently been reviewed in more detail (48). Nephrocytes are suitable to study the functional role of candidate genes that were identified in genetic studies and also elucidate the involved functional pathways which is facilitated by the low redundancy of the fly genome. The speed, cost-effectiveness, and reliability of this model make it well equipped to handle the considerable number of candidate variants that may result from contemporary sequencing efforts like whole exome sequencing. Beyond that, nephrocytes can also be applied to assess the significance of individual candidate mutations using transgenic rescue constructs. In the near future, *Drosophila* nephrocytes may further facilitate the development of targeted therapies and personalized medicine.

The most extensive work in *Drosophila* nephrocytes concerns steroid resistant nephrotic syndrome (SRNS). This disorder is characterized by childhood onset of edema, massive proteinuria, and progression to ESRD. SRNS is the second most frequent cause of ESRD within the first two decades of life and monogenic mutations explain a significant fraction of cases that manifest before 25 years of age (49). By now, more than 50 different genes have been implicated as a single gene cause for this disorder (50, 51) and more genes are likely to be discovered. The large number of known SRNS genes prompted systematic analyses that showed that about 60–80% of the studied orthologs of human disease genes resulted in a reduction of tracer uptake (7, 38) including genes that are involved in the slit diaphragm complex, actin regulation, CoQ_10_-biosynthesis, or interaction with the extracellular matrix. This bears testimony to the surprising extent of evolutionary conservation and underlines that central pathomechanisms of podocytopathies can be studied in nephrocytes. This is most obvious for *COQ2*-nephropathy (52) where findings in *Drosophila* indicate formation of reactive oxygen species as a critical event in the pathogenesis. Successful drug treatment of flies further provides evidence for Vanillic acid as a potential treatment (7). Importantly, these data further support that *COQ2*-nephropathy may be treatable (53) and that treatment strategies can be tested in nephrocytes. Some of these findings were confirmed in a recent manuscript that furthermore implicated autophagy and mitophagy as pathomechanistically relevant (54).

For the identification of several of the SRNS genes, studies in nephrocytes were instrumental by providing functional evidence supporting the genetic findings. This includes the KANK proteins (55), *ARHGDIA* (56), *ADCK4* (57), and most extensively *SGPL1* (58). For the ortholog of *SGPL1*, rescue constructs that reflect the mutations from SRNS patients were shown to be inefficient in contrast to a rescue with wild-type constructs. *Drosophila* nephrocytes also exhibited an altered lipid metabolism reflecting the observations in the SRNS patients.

### Diabetic Nephropathy

Diabetic nephropathy represents the single most significant cause of ESRD (1). Targeted therapies are unavailable and the current regimes are only sufficient to slow down the progress of disease. It is thus noteworthy that nephrocytes were found to be a model for diabetic nephropathy (59). High-glucose diet was shown to induce nephrocyte dysfunction and decrease the amount of Sns, which represents the ortholog of nephrin. The authors identified a transcriptional downregulation by a pathway that includes *Knot*, the ortholog of *EBF2*, to be responsible for the reduction of Sns in nephrocytes. Interestingly, the authors were able to validate their findings in *Drosophila* nephrocytes in mouse models of diabetic nephropathy.

### *APOL1-*Associated Nephropathies

Incidence of ESRD is nearly fourfold in African Americans compared with those of European descent. Much of the excess risk is attributable to two risk alleles of the *APOL1* gene. The study of *APOL1* is hampered by its poor evolutionary conservation as orthologs are lacking in most model organisms. Transgenic expression of the human *APOL1* in nephrocytes has been shown to result in a cellular toxicity that was more pronounced upon expression of the human *APOL1* risk alleles (44, 45). Nephrocyte dysfunction and loss was observed with increasing age of the flies, involving a mechanism connected to endosomal trafficking. More recently, the findings in *Drosophila* were confirmed by transgenic expression of the human gene in mouse (60). Essential features of the human disease were recapitulated in this model. It is intriguing, that the role of a common risk allele was studied successfully in *Drosophila* nephrocytes.

### Endocytosis and Proximal Tubular Diseases

Imerslund–Gräsbeck syndrome is characterized by Cobalamin-deficiency and tubular proteinuria while renal function is usually preserved. Mutations of Cubilin (61) and Amnionless (62) have been shown to be causative for this disorder. The orthologs of both genes are expressed in *Drosophila* nephrocytes, exercising an analogous role for nephrocyte function (28). Megalin, that may cause Donnai–Barrow syndrome (63) that also involves tubular proteinuria in humans, does not seem to be required for nephrocyte function (7, 27). A megalin-indepdendent role of Cubilin/Amnionless has also been described in humans for the absorption of vitamin B_12_ in the terminal ileum (64).

### Organ Crosstalk

*Drosphila* is an appropriate model to study communication between organs. In an effort to identify renal cardiomodulatory factors, Hartley and colleagues induced nephrocyte disruption and observed signs of a cardiomyopathy in the fly. Analyzing the hemolymph composition, they were able to identify *secreted protein acidic and cysteine rich* (SPARC), whose circulating levels are downregulated by nephrocytes. Reducing the gene dosage of this factor independently, through a heterozygous null allele, ameliorated the cardiomyopathy. Interestingly, *SPARC* has been linked to cardiac aging and metabolic syndrome in humans.

### Translational Applications

The genetic tool-kit of Drosophila combined with the cost-effectiveness and speed inherent to this model offer great opportunities for a translational approach. Only a glimmer of these opportunities can be recognized in the effect of Vanillic acid on defective CoQ_10_-biosynthesis (7). Screening platforms have been introduced that already enable large-scale genetic screens. These protocols need to be optimized to facilitate the discovery of treatment options for glomerular diseases.

## Outlook

We have seen significant progress in the last decade, and the nephrocyte currently emerges from the phase of experimental establishment to a driving force of glomerular discovery research. Combining a functional, accessible slit diaphragm with the power of the genetic tool-kit in *Drosophila*, the nephrocyte as a complementary model system is well equipped to reveal mechanisms of podocyte function and glomerular diseases. Once the enormous potential for translational applications is unlocked, the nephrocyte will play its role in the identification of targeted therapies that are urgently needed in nephrology.

## Author Contributions

The manuscript was written by TH with help from MH, MH designed the figures with help from TH, TBH critically reviewed and edited the manuscript and gave relevant additional insight. The title was suggested by TBH.

## Conflict of Interest Statement

The authors declare that the research was conducted in the absence of any commercial or financial relationships that could be construed as a potential conflict of interest.
